# Pregnancy outcome and risk of recurrence after tissue-preserving loop electrosurgical excision procedure (LEEP)

**DOI:** 10.1007/s00404-022-06760-5

**Published:** 2022-09-07

**Authors:** Jule Alena Lieb, Anne Mondal, Lenard Lieb, Tanja Natascha Fehm, Monika Hampl

**Affiliations:** 1 Clinic of Internal Medicine, Evangelisches Krankenhaus Oberhausen, Oberhausen, Germany; 2 Department of Gynecology and Obstetrics, University Hospital of Düsseldorf, Moorenstrasse 5, 40225 Düsseldorf, Germany; 3 School of Business and Economics, Maastricht University, Maastricht, The Netherlands

**Keywords:** LEEP, Conisation, Cervical intraepithelial dysplasia, Pregnancy outcome, Preterm birth, Premature rupture of membranes

## Abstract

**Background/purpose:**

This study aims to investigate whether women with cervical dysplasia after LEEP have an increased risk of pregnancy/childbirth complications or recurrence of dysplasia in an upcoming pregnancy.

**Methods:**

Data from 240 women after LEEP were analysed retrospectively. The reference group consisted of 956 singleton births. Fisher’s and Wilcoxon rank tests were used to detect differences between groups. Using logistic regressions, we analysed the effect of surgery-specific aspects of LEEP on pregnancy/childbirth complications and the frequency of CIN recurrences.

**Results:**

We found that tissue-preserving LEEP did not lead to premature birth or miscarriage and did not increase the likelihood of CIN recurrence.

We did not observe differences regarding preterm birth [< 37 (*p* < 0.28) < 34 (*p* < 0.31), < 32 weeks of gestation (*p* < 0.11)] or birth weight (< 2500 g (*p* < 0.54), < 2000 g (*p* < 0.77) between groups. However, women after LEEP exhibit a higher risk of premature rupture of membranes (PROM) at term (*p* < 0.009) and vaginal infections (*p* < 0.06). Neither volume nor depth of the removed tissue nor an additional endocervical resection seems to influence the likelihood of premature birth or early miscarriage. Performing an endocervical resection protects against CIN recurrence (OR 0.0881, *p* < 0.003).

**Conclusions:**

After tissue-preserving LEEP, there is an increased risk of vaginal infections and PROM at term in consecutive pregnancy. LEEP does not affect prematurity or miscarriage. The removal of additional endocervical tissue appears to be a protective factor against recurrence of CIN.

## What does this study add to the clinical work

**Table:** 

Colposcopically guided LOOP electrosurgical excision procedure helps to minimize the risk of prematurity in upcoming pregnancies in young women undergoing treatment of high grade CIN. Women should be offered this kind of treatment when further pregnancies are planned. The risk of recurrence of CIN is low.	

## Introduction

Human papillomavirus (HPV) infection is one of the most common sexually transmitted viral diseases. Almost 80% of all sexually active people become infected with HPV during lifetime. Most people clear infection. Only in cases with persistent infection with one of the high-risk HPV subtypes, cervical intraepithelial neoplasia (CIN) or/and carcinoma of the uterine cervix may develop. If advanced dysplasia (CIN3) is detected by cervical biopsy, Pap smear, and/or colposcopy, surgical treatment is recommended according to the German guidelines to prevent the development of cervical carcinoma [1]. The most commonly used surgical technique to treat high-grade CIN is colposcopically guided loop electrosurgical excision procedure (LEEP)

In the literature, LEEP has been shown to be associated with an increased risk of complications during pregnancy and birth. Most notably, preterm delivery by inducing preterm labour and preterm rupture of membranes (PROM) with consecutively low birth weight (< 2500 g) are often associated with this procedure [2–14]. Surgically removing dysplastic tissue from the uterine cervix means that uterine tissue, which could serve as support tissue in future pregnancies, is missing. This increases the risk of (preterm) premature rupture of membranes and cervical incompetence, and therefore, of premature birth and/or low birth weight. Furthermore, LEEP could further increase the risk of miscarriage and vaginal infections, since a surgical intervention can change the micro-environment of the uterine cervix [15–17].

To minimize these pregnancy- and birth-related risks, LEEP at the dysplasia unit at University Hospital of Düsseldorf (UKD) is performed in the child-bearing age group in a minimally invasive manner under colposcopic guidance to minimize the excised volume and damage to the cervix. Using this technique, only the colposcopically visible major change lesion is excised (with a LOOP) and the surrounding minor change ectocervical lesions are CO_2_-laser-vaporized. To prove the endocervical in sano resection histologically, an additional, small endocervical resection with a Mini-LOOP (0.5 cm) is performed in most cases. Finally, the wound will be CO_2_-laser-vaporized for complete haemostasis.

This tissue-preserving method, however, may influence the effectiveness of LEEP in treating high-grade CIN: a sufficient resection of dysplastic tissue is crucial to stop the dysplastic change from progressing and even prevent the development of cervical cancer and minimize the rate of recurrence.

The aim of this study is to investigate whether tissue-sparing LEEP increases the risk of premature birth, low birth weight, miscarriage, or recurrence of cervical dysplasia in a single-centre study of women treated in a large dysplasia unit in Germany.

## Patients and methods

**Fig. 1:**
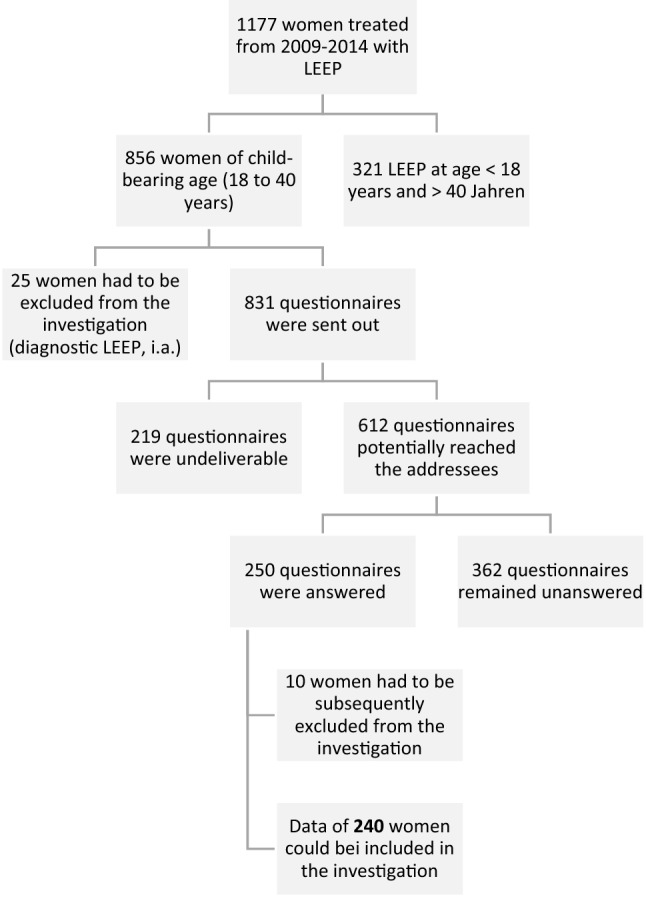
Overview of survey and final number of respondents

LEEP was performed in a total of 1177 women treated at the dysplasia unit at UKD from 2009 to 2014 (see Fig. 1).

Data of colposcopic findings and histological diagnoses by cervical biopsy, surgical procedure and histology, and depth and volume of excised cervical tissue were collected through our institution’s centralised medical computerised record system (Medico). 73% of women were between 18 and 40 years old, considered to be in child-bearing age. We sent out a survey to these former patients with specific questions concerning potential pregnancies and birth-associated complications, miscarriages, or recurrence of CIN. Moreover, the questionnaire included questions related to information about numerous other variables which might be relevant to control for in a statistical analysis, such as health conditions, previous pregnancies, and previous preterm deliveries (amongst others). The institutional ethic board of the hospital approved all studies linked to the study and patients agreed to the analyses of their data being informed consent.

Among the women who responded to the questionnaire, 91 women gave birth to a singleton after undergoing tissue-sparing LEEP at UKD. We used a sample of 956 (non-LEEP) singleton births, documented at UKD from January to June 2016 as a reference group. Information about individuals in our reference group included a list of possibly relevant covariates which we used in the regression analysis.

Based on these data, we analysed two questions.

(i) *Are there differences regarding important outcome variables between patients that underwent (tissue-preserving) LEEP and those who did not?*

Next to simple descriptives, Fisher’s exact test and the Wilcoxon signed-rank tests were used to examine whether there were differences between the study and reference group. Furthermore, we performed several logistic regression analyses to quantify the effect of (tissue-preserving) LEEP on important outcome variables. In all regression analyses, LEEP was included as a categorical regressor variable (i.e., a zero–one dummy), and the set of covariates observed for both groups were included as controls.

(ii) *What are the effects of surgery-specific aspects of LEEP (depth of conus/volume of the excised cervical tissue, removal of additional endocervical tissue, *etc*.) on pregnancy or birth complications as well as on the recurrence rate of CIN?*

We used logistic regressions to estimate quantitative effects. The regression analysis was based on the data for the treatment group only. For that group, a large set of possibly relevant control variables was collected through the survey. Including all variables as regressors would increase the degrees of freedom drastically relative to the number of observations, which in turn would result in imprecise estimation and inference. This forces us to explicitly consider model selection to select only those controls that are “most relevant”. For every regression, we used the post-double model selection procedure as proposed in Belloni and Chernozhukov [18]. The results of the regression analyses are expressed in odds ratios (OR). 95% confidence intervals (CI) are reported, as well. *P *values are reported for regression coefficients as well as for all conducted tests. The program ‘R’ was used for statistical analyses.

## Results

*Gestational age and birth weight* The mean gestational age in our treatment group (*n* = 91) women with singleton birth after LEEP) was 38.96 weeks of gestation (SD 1.99 weeks; median = 39 weeks). The rate of premature births was 9.89%. 2.2% of the pregnancies ended before 34 weeks of gestation. The mean birth weight at delivery in our treatment group was 3370.22 g (SD 526.52 g; median = 3350 g).

In our reference group (*n* = 956), women delivered her baby on average at 38.19 weeks of gestation (SD 2.5 weeks; median = 39 weeks). The rate of premature births was 14.12%. 4.81% of pregnancies ended before 34 weeks of gestation. The mean birth weight in the reference group was 3275.63 g (SD 609.41 g; median = 3330 g) (see Table 1).

**Table 1:** Summary of statistics for the study (*n* = 91) and reference group (*n* = 956)

	Study group (*n* = 91)	Reference group (*n* = 956)	*p* (Fisher’s exact test)
Mean gestational age at delivery (weeks)	38.96	38.19	–
Preterm delivery	9.89%	14.12%	0.27
Delivery < 34 weeks	2.2%	4.81%	0.306
Delivery < 32 weeks	0.0%	2.82%	0.103
Mean birth weight at delivery (g)	3370.2	3275.63	–
Birth weight at delivery median (g)	3350.0	3330.0	–
Low birth weight (< 2500 g)	5.49%	7.74%	0.539
Low birth weight (< 2000 g)	2.20%	3.45%	0.763
PROM or cervical incompetence	44.44%	17.04%	0.008
Vaginal infections	4.4%	1.36%	0.053
Spontaneous delivery	50.55%	52.30%	–
Caesarean section rate	35.16%	41.63%	0.265
Vacuum/forceps delivery	14.29%	6.07%	0.007

We did not find statistically significant differences in premature births [< 37 (*p* < 0.28), < 34 (*p* < 0.31), < 32 weeks of gestation (*p* < 0.11)] and low birth weight [< 2500 g (*p* < 0.54), < 2000 g (*p* < 0.77)] between the treatment group and reference group (see Table 1). 

Moreover, we did not find evidence that the likelihood of a premature birth is affected by the depth [OR 1.1591, 95% CI (0.06–22.72), *p* < 0.93] or the volume [OR 0.6516, 95% CI (0.29–1.44), *p* < 0.3] of the removed cervical tissue, nor by the excision of additional endocervical tissue [OR 1.2606, 95% CI (0.09–17.67), *p* < 0.87] (see Table 2).

**Table 2:** Selected regression results showing effect, in terms of odds ratios, of surgery-specific regressors (columns) on various outcome variables (rows)

	Depth	Volume	Removal of additional endocervical tissue
Premature birth	OR 1.1591 95% CI (0.06–22.72) *p* = 0.9226	OR 0.6516 95% CI (0.29–1.44) *p* = 0.2901	OR 1.2606 95% CI (0.09–17.67) *p* = 0.8636
Miscarriage	OR 2.4197 95% CI (0.15–38.80) *p* = 0.5325	OR 0.3797 95% CI (0.06–2.38) *p* = 0.3007	OR 4.6615 95% CI (0.30–71.47) *p* = 0.2691
Recurrence	OR 0.3467 95% CI (0.008–15.78) *p* = 0.5863	OR 0.5037 95% CI (0.096–2.66) *p* = 0.4188	OR 0.0811 95% CI (0.016–0.41) *p* = 0.0024

Further controls were included

Estimation results are not shown* Pregnancy-associated complications:* Based on Fisher’s exact test, we found a significant difference between LEEP patients and the reference group regarding premature rupture of membranes (PROM) (*p* < 0.009) at term and vaginal infections in subsequent pregnancies (*p* < 0.06) (see Table 1). Estimates from a logistic regression analysis indicated a significantly increased chance of PROM [OR 3.8904, 95% CI (1.56–9.69), *p* < 0.004] due to LEEP. Similarly, the occurrence of preterm premature rupture of membranes (PPROM) also significantly increases after undergoing LEEP [OR 13.8427, 95% CI (2.03–94.37), *p* < 0.008; see Table 3], but not leading to preterm deliveries (see above).

**Table 3:** Selected regression results showing effect, in terms of odds ratios, of LEEP on various birth-specific outcome variables. Further controls were included; estimation results are not shown

	LEEP
Preterm delivery	OR 0.3833 95% CI (0.16–0.93) *p* = 0.0344
Low birth weight (< 2500 g)	OR 0.662 95% CI (0.25–1.74) *p* = 0.4038
Low birth weight (< 2000 g)	OR 0.6082 95% CI (0.14–2.70) *p* = 0.5128
PROM	OR 3.8904 95% CI (1.56–9.69) *p* = 0.0035
PPROM	OR 13.8427 95% CI (2.03–94.37) *p* = 0.0073

*Mode of delivery* Fisher’s test showed no statistically significant difference between the treatment and reference groups when assessing the frequency of caesarean sections (*p* < 0.27). In our study group, more women had a forceps delivery (14.29% versus 6.07%; OR 2.58, *p* < 0.008).

*Miscarriage* The rate of miscarriages after LEEP was 11.88% in our treatment group. Neither the depth [OR 2.4197, 95% CI (0.15–38.80), *p* < 0.54], nor the volume [OR 0.3797, 95% CI (0.06–2.38), *p* < 0.31] of the removed tissue, nor an additional endocervical resection [OR 4.6615, 95% CI (0.30–71.47), *p* < 0.27] affected the likelihood of miscarriage (see Table 2). Our results confirm findings in the literature that a prior miscarriage increases the risk of a further miscarriage after LEEP [OR 7.6067, 95% CI (1.18–49.08), *p* < 0.04].

*Recurrence of CIN* After undergoing LEEP at UKD, 4.17% of patients developed a CIN recurrence requiring further cervical surgery. Neither the depth [OR 0.3467, 95% CI (0.008–15.78), *p* < 0.59] nor the volume [OR 0.5037, 95% CI (0.096–2.66), *p* < 0.42] of the removed cervical tissue affected the need for further cervical surgeries. Neither the severity of CIN [OR 1.808, 95% CI (0.43–3.26), *p* < 0.75], nor the histological status of the ectocervical margins (*p* < 0.87), nor the histology of endocervical margin (*p* = 0.99) increased the need of further surgeries. Removing additional endocervical tissue appears to protect against the recurrence of CIN [OR 0.0811, 95% CI (0.016–0.41), *p* < 0.003] (see Table 2).

## Discussion

The frequency of premature births post-LEEP conization varies widely in the current literature [2–14]. For example, the meta-analysis by Jin et al. showed that LEEP is associated with an increased risk of premature birth. The relative risk of preterm delivery was 1.84, and the relative risk of giving birth to a child < 32/34 weeks of gestation was 1.98 [3]. Maina et al. investigated premature birth after LEEP and/or laser conization, finding that the proportion of premature birth was even 33.13% versus 6.60% in the control group (*p* < 0.0001) [4].

In our study, we did not find that premature births are more likely after tissue-preserving LEEP compared to our control group (9.89% vs. 14.12%). Note also that the average premature birth rate in our treatment group after LEEP is close to the nationwide premature birth rate in Germany (8.64% in 2016) [19]. Moreover, the distributional characteristics of birth weight in our treatment group are in line with those found in the wider population. In 2015, the proportion of newborns with a birth weight below 2500 g in Germany was 6.6% [20]. In our study, 5.49% of newborns had a birth weight < 2500 g when mothers had LEEP for high-grade CIN before their pregnancy.

The influence of the depth/volume of the removed tissue is also discussed in the literature, and some studies have shown a negative correlation between the depth/volume of the removed tissue and the gestational age at birth [21–23].

Noehr et al. showed that the greater the cone thickness, the greater the risk of premature birth. The authors found a 6% increase in risk with every additional millimeter of cervical tissue removed [OR 1.06, CI (1.03–1.09)] [21]. Similarly, Liverani et al. found an inverse correlation between cone length and gestational age at birth (*p* < 0.001) [23].

In our analysis, we did not find a connection between the extent of the excision of cervical tissue and the gestational age. This may be due to the (cervical) tissue-sparing manner in which LEEP is performed in our dysplasia unit, where only the excision of the major change lesion is performed with a size-adapted loop and the surrounding minor change lesion is removed with laser vaporization.

We additionally examined complications directly leading to prematurity. In the treatment group, PPROM or cervical incompetency occurred in 44.44% of our cases. In contrast, only 17.04% of the women in our reference group affected by preterm delivery suffered from PPROM. Our regression analyses confirmed an increased risk for PROM and PPROM after undergoing LEEP even if it is done in a tissue-sparing manner. These findings are similar to those in the literature, where the risk for (P)PROM is also found to be increased after LEEP [3, 4, 8, 10, 25]. Maina et al. found an increased proportion of PROM in the study group after LEEP compared to the control group (40.00% versus 23.22%) [4]. Wittmaack et al. found that that a conisation may trigger the occurrence of PPROM [OR 276.02, 95% CI (101.47–750.83), *p* < 0.001] [8]. Armarnik et al. (2011) found that the rate of PPROM is more than twice as high after conization (15.1% versus 7.1%) [10]. In the meta-analysis by Jin et al., the relative risk of PPROM was 2.91 (RR = 2.91, *p* < 0.0001) [3]. This means that women who give birth to a child after LEEP have an almost threefold higher risk of PPROM.

The risk of vaginal infections may be higher following LEEP, since the micro-environment of the uterine cervix is altered by the surgical intervention. The mucus composition and vaginal microbiome (cytokines, etc.) may change after conization, since a portion of the glands has been surgically damaged or removed. In addition, scar tissue can form after surgical intervention [15–17]. All these LEEP-related factors can foster a vaginal infection. We indeed find evidence for an increased chance of vaginal infection during pregnancy for women that underwent a tissue-preserving LEEP.

The birth mode can also be affected: after performing LEEP, the uterine cervix could become scarred and the structural changes could result in loss of elasticity of the cervix. Both factors may make a natural birth more difficult. In our study, however, we did not find evidence that LEEP patients are more likely to require a caesarean section compared to our reference group (see Table 1).

11.88% of the women in our treatment group who became pregnant for the first time after cervical surgery suffered an early miscarriage. This is close to the lower boundary of the miscarriage rate in the general population, estimated to be between 11 and 31% [25, 26].

We found that neither the depth nor the volume of the excision does affect the likelihood of a miscarriage. This again may be due to the minimally invasive, colposcopically guided manner in which LEEP is performed in our institution. Leiman et al. found that the depth of the removed tissue correlates with pregnancy-related complications and showed that the risk of miscarriage increases in direct proportion to the thickness of the removed tissue [27]. Since these data are from 4 decades ago, the surgical technique was probably quite different and is responsible for these results. Khalid et al. also found an increased risk of miscarriage, especially if the thickness of the excision exceeded 12 mm [28]. In our treatment group, the proportion of excised tissue with thickness exceeding 12 mm was only 10%.

LEEP in a colposcopically guided manner in women in child-bearing age aims to minimize the damage caused to the cervical tissue to minimize complications for further pregnancies. This strategy may, however, may have a negative influence on the effectiveness and oncologic safety of LEEP in treating CIN3: a sufficient resection of the dysplastic tissue is crucial to stop the dysplastic change from progressing and even prevent CIN3 from developing into cervical cancer.

Arbyn et al. argue that patients with a history of CIN are, despite treatment, at higher risk of developing invasive carcinoma compared to the general population [29]. Only 4.17% of the patients in our treatment group had to undergo a further operation on the uterine cervix because of CIN recurrence. In contrast, Verguts et al. found an 8% recurrence rate of CIN [30]; Pires et al. found a recurrence of CIN in 13.3–16.2% of cases after performing LLETZ [31]. In a study by Xi et al., CIN2-3 was detected in 10% of the cases within 2 years after performing LEEP with an initial CIN3 diagnosis [32].

Note that, in contrast to other studies, our data allow us to draw conclusions about long(er)-term effects. The operations were performed between 2009 and 2014, and the patients were contacted in December 2015/January 2016. Thus, our results concerning recurrence of CIN cover a period of up to 7 years post-surgery.

Our finding that the removal of additional endocervical tissue significantly lowers the risk of recurrence of CIN is noteworthy. We also investigated whether the removal of additional endocervical tissue increases the risk of a premature birth. To our knowledge, this aspect has not yet been considered in the literature. We found that the removal of additional endocervical tissue does not increase the occurrence of prematurity. Nevertheless, endocervical resection may not be necessary in every case, especially if the major change lesion is easily visible and (only) located ectocervically, but rather indicated when an endocervical part of a major change lesion is suspected. This aspect serves to avoid unnecessary removal of healthy tissue.

## Conclusions

We did not find evidence that colposcopically guided tissue-preserving LEEP affects prematurity or miscarriage. There is an increased risk of vaginal infections and (preterm) premature rupture of membranes. Despite the tissue-sparing implementation of LEEP in our dysplasia unit in women in the child-bearing age, the risk of CIN recurrence proves to be very low. The removal of additional endocervical tissue appears to constitute a protective factor against recurrence.

## Abbreviations

CI: Confidence interval
CIN: Cervical intraepithelial neoplasia
g: Gram
HPV: Human papilloma virus
LEEP: Loop electrosurgical excision procedure
OR: Odds ratio
PPROM: Preterm premature rupture of membranes
PROM: Premature rupture of membranes
SD: Standard deviation
UKD: University Hospital of Düsseldorf
